# Analysis of the variation in potential evapotranspiration and surface wet conditions in the Hancang River Basin, China

**DOI:** 10.1038/s41598-021-88162-2

**Published:** 2021-04-21

**Authors:** Han Zhang, Lin Wang

**Affiliations:** College of Environmental Science and Engineering, Key Laboratory of Marine Environment and Ecology, Ministry of Education, Ocean University of China, Qingdao, 266100 China

**Keywords:** Hydrology, Environmental impact

## Abstract

Evapotranspiration is an important component of the water cycle, and possible trends in evapotranspiration can, among others, influence water management and agricultural production. Potential evapotranspiration (ET_p_) is a measure of the ability of the atmosphere to remove water from the surface through the processes of evaporation and transpiration. It plays an important role in assessing regional dry–wet conditions and variations in meteorological conditions. This study analyzed the change trends of monthly ET_p_ and surface dryness and wetness in the Hancang River Basin and, through principal component analysis and correlation analysis, explored the main meteorological factors that affected ET_p_ and the interactions between meteorological factors; ET_p_ values were estimated using the FAO-56 Penman–Monteith method. The results showed that there was a large gap in ET_p_ between different months in the Hancang River Basin, with a trend of first increasing and then decreasing within a year. The highest monthly evapotranspiration was 114.119 mm (July), and the lowest was 42.761 mm (January). The maximum relative humidity index was 0.822 (August), and the minimum was -0.979 (January). The average temperature, precipitation, average relative humidity, and solar radiation are positive factors that affect ET_p_, while average air pressure is a negative factor that affects ET_p_. This study provides a reference for the wet conditions of small watersheds and for countermeasures to address climate change.

## Introduction

Since worldwide mean global surface temperatures have increased significantly in recent decades^1,2^, changes in other components of the hydrological cycle and its processes can also be expected^3^. A warming climate is expected to have a significant influence on water storage and consequently, on worldwide water availability^4,5^. Evaporation occupies an extremely important position in the hydrological cycle and is closely related to the amount of surface water and energy balance^6,7^. Increased evapotranspiration could lead to decreased surface and subsurface runoff, decreased groundwater storage and consequently, to water management issues^8^. The reasons for the changes in evaporation are generally believed to be related to climatic conditions and ecological factors in different regions. Rising temperatures will increase water evaporation and surface evaporation. Direct factors which affect evaporation in arid areas are light and moisture^9–11^. Therefore, in the context of global warming and drying, it is imperative to strengthen research on the distribution characteristics and regional climate elements.

Evapotranspiration is an important component of the water cycle and energy balance and hence, drives the Earth’s climate system at various scales^12,13^. Evapotranspiration can be regarded as one of the most important factors for indicating climate change at the catchment scale since it influences both surface runoff and water storage in catchments^8,12^. The phenomenon of evaporation in nature is quite complicated. Due to the lack of measured evaporation data, estimations of regional ET_p_ in hydro meteorological research have always been difficult. Therefore, reference evapotranspiration values are estimated by using empirical or combination (energy and mass transfer) methods^14^. Among these, combined methods require only daily air temperature and radiation data; empirical methods require daily data for air temperature, solar radiation, vegetative canopy, wind speed, and relative humidity and the calculation process is relatively complicated, such as for FAO56-PM model; this model is widely used and is of great importance for evaluating regional wet conditions, crop water requirements, and water resources management^15,16^. Numerous studies have previously been conducted to examine trends in reference evapotranspiration such as the work of Azizzadeh and Dadaser-Celik, who defined atmospheric evaporative demand of a reference crop which is estimated based on climatic variables^17,18^. It was found that evapotranspiration trends vary significantly with climatic conditions and across regions^19,20^. Thompson et al. studied the changing ET_p_ trends in the Mekong River Basin^21^. Luab et al. analyzed the changing characteristics and influencing factors of evapotranspiration and demonstrated evapotranspiration trends in different seasons by using the multiple linear regression analysis method and the main factor weighted comprehensive^22^. To date, relevant studies have focused on the temporal and spatial evolution trends of evapotranspiration, the ecological elements that affect the change in evapotranspiration, and the correlations between evapotranspiration and meteorological factors^23,24^. Additionally, drought and water shortages are the main challenges to resources and ecology and climate change will have a great impact on the hydrology of a region^16^. Therefore, studying the impact of climate on regional ET_p_ is helpful for understanding the response of climate change characteristics to regional hydrological cycles.

The evapotranspiration process depends on vegetation characteristics, climate variables and environmental factors and according to the parameters and definitions of the reference evapotranspiration model, it is found that the only factors that affect the changes in reference evapotranspiration are climate variables^8,15^. Many researchers have also studied the causes for the change in evapotranspiration across different regions of the world. However, earlier results have indicated that the significance of climatic variables influencing reference evapotranspiration vary from region to region^12,20,25^. Cesar and Šraj provided an overview of the influencing factors and methods for calculating reference evapotranspiration rates and conducted a sensitivity analysis of climatic factors affecting reference evapotranspiration^26^; their results showed that reference evapotranspiration values mainly depended on solar radiation which was followed in importance by air temperature and relative humidity while wind speed had the least influence. Adnan et al. analyzed changes in seasonal and annual evapotranspiration from 1951 to 2016 at 50 meteorological stations which were located in extremely arid, arid, and semi-arid zones of Pakistan using the Penman–Monteith (PM) method and found that evapotranspiration was positively correlated with temperature, solar radiation, and wind speed and was negatively correlated with air pressure^27^. Duethmann and Blöschl conducted a statistical analysis of reference evapotranspiration in 156 Austrian watersheds and concluded that the main reasons for increased reference evapotranspiration were the increase in net radiation and rise in temperature^28^. On the other hand, Yang et al. pointed out that the main parameter for evapotranspiration in the Yellow River Basin was relative humidity which was followed in importance by average temperature, solar radiation and wind speed^12^. Furthermore, Wang et al. studied the effect of climate change on evapotranspiration in the Hetao irrigation area and pointed out that evapotranspiration was most sensitive to average daily temperature and was followed in importance by wind speed and average relative humidity^20^. In summary, the change of ET_p_ is a combined result of many factors. But the dominant factor is different in different regions and at different scales. At present, research of evapotranspiration and meteorological elements is concentrated in large areas, such as countries^8^, provinces^14^, and watersheds^29^ while research on evapotranspiration in small watersheds is relatively rare and the research methods and systems used are not standardized. In addition, there are few literature studies on the seasonal and monthly variation trend analysis of ET_p_ in temperate monsoon regions with significant dry and wet climates and the main meteorological factors affecting ET_p_. The important components of the hydrological cycle and the impact of climate change on ET_p_ are also unclear. The law of changes in the surface dry and wet conditions has not been mentioned. Therefore, it is necessary to create new methods for the study of evapotranspiration in small basins and the surface dry and wet conditions.

Accordingly, the objectives of this study are: Based on meteorological observation data and precipitation data in the Hancang River Basin, the Penman–Monteith formula [recommended by the World Food and Agriculture Organization (FAO)] is used to calculate monthly basin ET_p_ values and relative humidity indexes. In this study, wavelet analysis, principal component analysis, correlation analysis and partial correlation analysis were used to explore the changes of ET_p_ and surface wet conditions in the Hancang River Basin and the main meteorological factors affecting ET_p_ and their interactions with various meteorological factors were analyzed. Additionally, our focus is on changes of monthly ET_p_ in the Hancang River Basin and their interactions with the relative humidity index and various meteorological factors.

## Data and methods

### Study site and data

**Figure 1:**
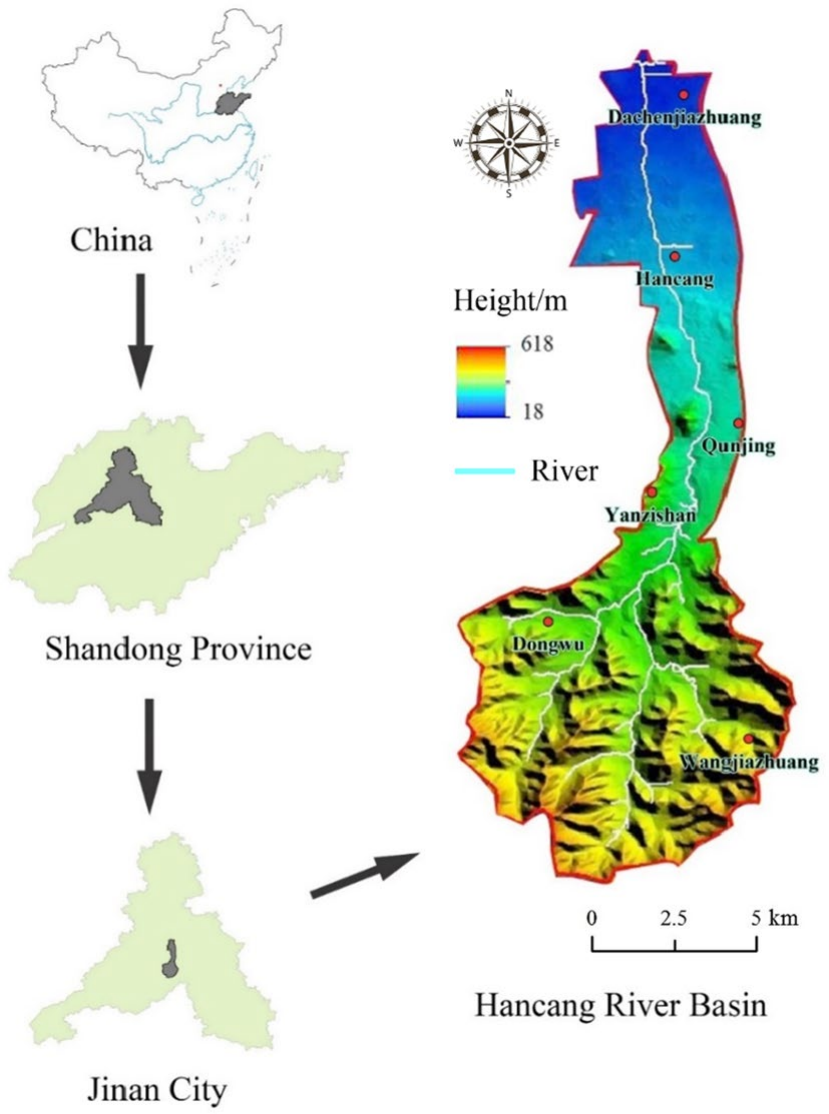
Location of study area. (China and Shandong Province data from Ministry of Natural Resources of China, http://www.mnr.gov.cn/; Jinan City and Hancang River Basin was generated by ArcGIS 10.3 software, https://desktop.arcgis.com/en/).

As shown in Fig. 1, the study area is the Hancang River Basin. The basin area is 100 km^2^, which indicates a small basin. The Hancang River is located southeast of the Licheng District, Jinan City and is a tributary of the Xiaoqing River. The Hancang River is a naturally formed rain-sourced flood drainage river in the southern mountainous area. The total length of the main stream is approximately 27.8 km and the basin mainly belongs to a warm temperate monsoon climate with four distinct seasons; the annual average temperature is 13.3 °C and average precipitation is 783.27 mm. The average precipitation from June to September accounts for 60.26% of the annual average precipitation and precipitation is greatest from July to August. The river has steep slopes and rapid flows, its maximum elevation difference is 339 m, it receives concentrated precipitation, and is prone to flooding.

First, based on the Arc Hydro Tools module in ArcGIS and Digital Elevation Model (DEM) data with a resolution of 30 m, the hydrological characteristics of the basin were extracted sequentially through flow direction analysis, confluence analysis, river network generation, and watershed boundary division. The DEM data were obtained from the China Geospatial Data Cloud Platform (http://www.gscloud.cn/).

Moreover, the observational data in this study included monthly average temperature, average pressure, average relative humidity, air density, average wind speed, and solar radiation of the basin over the past 30 years which was provided by China Meteorological Science Data Center (http://data.cma.cn/) and Jinan Meteorological Bureau (http://sd.cma.gov.cn/).

In this study, the meteorological data were obtained from 6 stations from 1989 to 2019 in the Hancang River Basin. The observation sites included Yanzishan, Dongwu, Hancang, Wangjiazhuang, Qunjing and Dachenjiazhuang. The locations of these stations fairly captures the different microclimate conditions of the region since each location of the six is found in a unique microclimate regime. Among them, Dachenjiazhuang and Hancang are located in the northern part of the basin; Yanzishan and Qunjing are located in the middle of the basin; and Dongwu and Wangjiazhuang are located in the southern part of the basin. The observation data for precipitation in the basin from the Jinan Hydrological Station, Shandong Province. The locations of stations are shown in Table 1.

**Table 1:** Weather station locations and climate type.

Station	Longitude	Latitude	Elevation (m)	Climate type
Yanzishan	117°03′E	36°39′N	101	Temperate monsoon
Dongwu	117°01′E	36°36′N	201	Temperate monsoon
Hancang	117°11′E	36°43′N	53	Temperate monsoon
Wangjiazhuang	117°19′E	36°31′N	251	Temperate monsoon
Qunjing	117°18′E	36°41′N	96	Temperate monsoon
Dachenjiazhuang	117°15′E	36°50′N	23	Temperate monsoon

### Methods

#### FAO Penman–Monteith method

Potential evapotranspiration (ET_p_) is defined as the evapotranspiration rate from the reference surface, which is a hypothetical grass with height of 0.12 m, albedo of 0.23 and surface resistance of 70 s/m. The FAO Penman–Monteith equation has been recommended as the sole standard method for determining ET_p_ since 1990 by the Food and Agricultural Organization of the United Nations (FAO)^15^. Air temperature, relative humidity, solar radiation and wind speed data are required when using this combined method which allows estimation of ET_p_ even in the case of missing climatic variables and different climatic conditions. Daily ET_p_ values were calculated according to the FAO Penman–Monteith equation^15^.

$$ET_{p} = \frac{{0.408\Delta \left( {R_{n} - G} \right) + \gamma \frac{900}{{T_{mean} + 273}}U_{2} \left( {e_{s} - e_{a} } \right)}}{{\Delta + \gamma \left( {1 + 0.34U_{2} } \right)}}$$

*Rn* is the net radiation at the crop surface (MJ m^−2^ day^−1^), *G* is the soil heat flux density (MJ m^−2^ day^−1^), *T* is the mean daily air temperature at 2 m height (°C), U_2_ is the wind speed at 2 m height (ms^−1^), *e*_*s*_ is the pressure (kPa), *e*_*a*_ is the actual vapor pressure (kPa), Δ is the slope of the vapor pressure curve (k Pa °C^−1^), and γ is the psychrometric constant (k Pa °C^−1^)^15^.

Net radiation (*Rn*) was calculated as the difference between incoming net shortwave radiation and outgoing net longwave radiation and followed the procedure of Allen et al. based on global solar radiation, albedo (0.23), clear-sky solar radiation, *T*_*min*_, *T*_*max*_ and *e*_*a*_^15^. Based on the same guidelines, the magnitude of daily soil heat flux (G) beneath the reference grass surface is relatively small and therefore, may be neglected for 24-h time steps^28,30,31^.

#### Wavelet analysis

Wavelet transform analysis explains many changes which are obscured in time series. Fast wavelet analysis does not involve specific wavelet functions or scaling functions and the calculations are fast and simple^32^. In this study, the wavelet transform method was used to analyze monthly ET_p_ values in the basin over the past 30 years and Origin software was used to calculate wavelet coefficients and draw the 3D bar charts and heatmap (https://www.originlab.com/).

#### Principal component analysis

The statistical method of principal component analysis of IBM SPSS Statistics 22 software was used to analyze the climate factors which influenced ET_p_ (https://www.ibm.com/analytics/spss-statistics-software). The main principle of principal component analysis is to investigate the correlations between multiple variables and reveal the internal structure of multiple variables through use a few principal components. This method is often used in multivariate analysis.

#### Correlation analysis and partial correlation analysis

Correlation analysis is a statistical method used to measure the closeness of two variables. Partial correlation analysis refers to the process of removing the influence of a third variable when two variables are related to the third variable at the same time and only analyzing the degree of correlation between the other two variables. The judgment index is the R value of the correlation coefficient. The calculation formula of the correlation coefficient is as follows:

$$R_{xy} = \frac{{\mathop \sum \nolimits_{i = 1}^{n} \left[ {\left( {x_{i} - \overline{x}} \right)\left( {y_{i} - \overline{y}} \right)} \right]}}{{\sqrt {\mathop \sum \nolimits_{i}^{n} \left( {x_{i} - \overline{x}} \right)^{2} } *\sqrt {\mathop \sum \nolimits_{i = 1}^{n} \left( {y_{i} - \overline{y}} \right)^{2} } }}$$

where *Rxy* is the correlation coefficient between variables *x* and *y*; *x*_*i*_ is the ET_p_ value of the month *i* (mm); *y*_*i*_ is the value of the climate factor in the month *i*, $\overline{x}$ and $\overline{y}$ are the monthly averages of ET_p_ and climate factors, respectively.

The calculation formula of partial correlation coefficient is as follows:

$$R_{xy,z} = \frac{{R_{xy} - R_{xz} *R_{yz} }}{{\sqrt {\left( {1 - R_{xz}^{2} } \right)\left( {1 - R_{yz}^{2} } \right)} }}$$

where *Rxy*, *z* are the partial correlation coefficients between the dependent variable x and the independent variable y after the independent variable z is fixed. The *T* test method is used to test the significance of the partial correlation coefficient, and the calculation formula is as follows:

$$t_{p} = \frac{{R_{xy,z} }}{{\sqrt {1 - R_{xz}^{2} } }}\sqrt {n - m - 1}$$

where *t*_*p*_ is the statistical quantity for the significance test of the partial correlation coefficient; *Rxy, z* are the partial correlation coefficients, *n* is the sample size, and *m* is the number of independent variables.

### Relative wetness index

Quantitative analysis of the relative humidity index reveals the characteristics of changes in wet conditions in the study area. In this paper, the relative humidity index recommended by the Chinese national standard “Meteorological Drought Grade (GB/T20481-2017)” is used to quantitatively analyze the change characteristics of dry and wet conditions in the study area^33^:

$$M = \left( {\frac{P - PE}{{PE}}} \right)$$

where *M* is the relative humidity index, *P* is the precipitation in a certain time period (mm), and *PE* is the ET_p_ in a certain time period (mm).

## Results

### Investigation of changes in ET_p_

**Figure 2:**
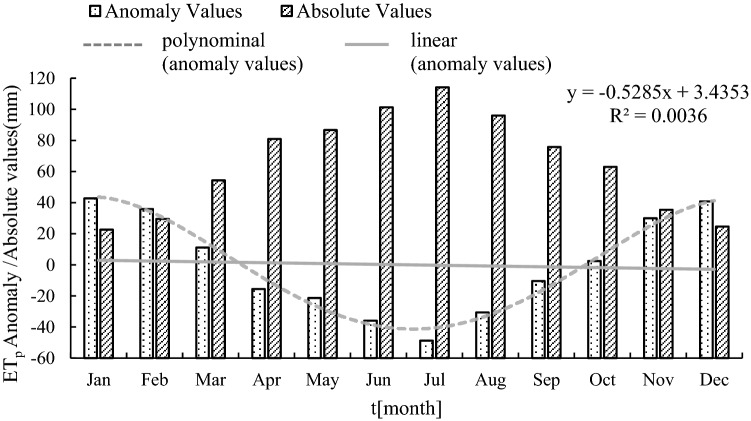
The anomaly and absolute value of ET_p_ in Hancang River Basin.

Figure 2 shows the following: The annual ET_p_ in the Hancang River Basin is 784.143 mm, the smallest is 22.584 mm (January), the largest is 114.119 mm (July), and the monthly average is 65.345 mm. From an overall point of view, ET_p_ anomaly values are significantly higher in winter than in summer and show a trend of first decreasing and then increasing throughout the year with the maximum value in January (42.761 mm) and the minimum value in July (− 48.773 mm). This shows that ET_p_ was lowest in January and reached the highest value in July. The fourth-order polynomial curve shows that ET_p_ fluctuates regularly in the Hancang River Basin. The ET_p_ anomalies from April to September are negative and anomalies in the remaining months are positive. Among them, changes in ET_p_ are most significant from February to March and from September to October.

**Figure 3:**
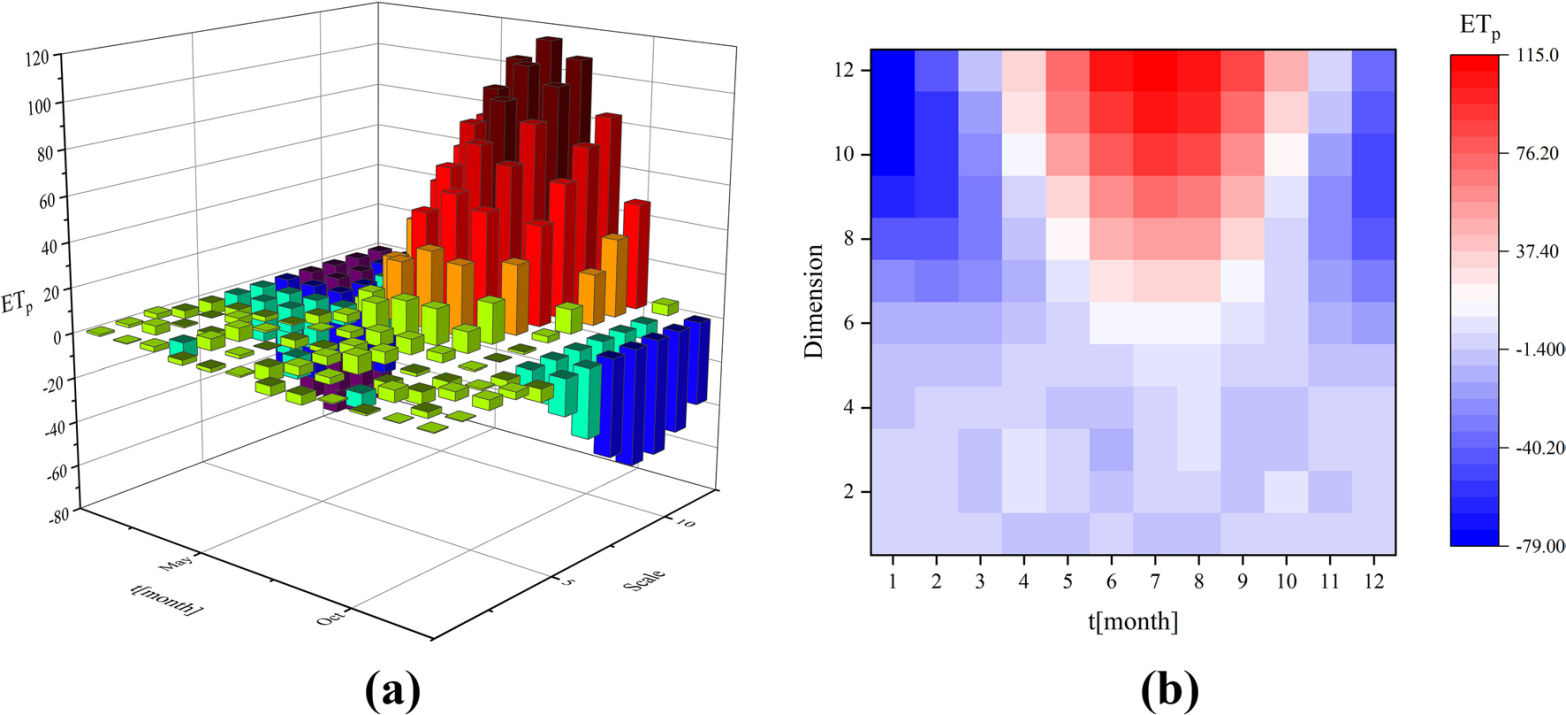
Wavelet transform 3D Bars and real part time–frequency distribution Haetmap for Hancang River Basin. (The figure was generated by Origin 2019b software, https://www.originlab.com/).

The wavelet transform analysis method was used to evaluate monthly ET_p_ values in the Hancang River Basin (Fig. 3). The wavelet 3D bars show that monthly ET_p_ values exhibit strong, abrupt changes, a significant monthly gap, and long duration in the Hancang River Basin (Fig. 3a). This is due to the significant effects of the monsoon climate, in which precipitation varies greatly in a seasonal manner. At this time, potential evaporation levels increase sharply in early summer and decrease in early autumn. The real time–frequency heatmap reveals the three-segment clustering centers and shows a trend from weak to strong and then to back to weak (Fig. 3b). The first dispersion center is near January and the time-domain scale strong concentration influence range is 8–12. The second is the strong concentration center near July where the time-domain scale strong concentration influence range is 7–12. The third is scattered around December, with a strong concentration in the time domain in the range of 6–12.

### Relationship between ET_p_ and climatic variables

First, principal component analysis of monthly weather data for the Hancang River from 1989 to 2019 was conducted. Table 2 shows the results of principal component analysis for this time period. The results indicate statistically that the dependence between the influencing factor variable and the original variable has remained at approximately 94% and that the variance contribution rate of the first principal component reaches 72.198%.

**Table 2:** Principal component analysis of meteorological factors in Hancang river basin.

Ingredient	Initial eigenvalue			Extract the sum of squares of the load		
	Eigenvalues	Variance contribution rate (%)	Cumulative contribution rate (%)	Eigenvalues	Variance contribution rate (%)	Cumulative contribution rate (%)
1	5.05387	72.19818	72.19818	5.05387	72.19818	72.19818
2	1.61375	23.05352	95.25170	1.61375	23.05352	95.25170
3	0.22272	3.18178	98.43348	0.22300	3.18200	98.43300
4	0.07020	1.00286	99.43634			
5	0.02929	0.41848	99.85482			
6	0.01012	0.14458	99.99940			
7	0.00004	0.00060	100.00000			

As shown in Table 2, the first principal component contains average temperature, precipitation, solar radiation, air density, and average air pressure. The variance contribution rate is 72.198%. The average temperature, precipitation, and solar radiation are distributed in the positive direction while air density and average air pressure are distributed in the negative direction. Therefore, when ET_p_ increases, the average temperature, precipitation, and solar radiation increase while the air density and average air pressure decrease. Therefore, it is speculated that the increase in temperature is the main factor for the increase of ET_p_ in the Hancang River Basin. The second principal component integrates average relative humidity and can reflect the influence of humidity on ET_p_ to a certain extent; annual increases in ET_p_ will inevitably affect the changes in wet and dry conditions of the basin. The third principal component consists of the average wind speed which is second only to temperature. Winds transfer and exchange carbon dioxide, oxygen, and heat and accelerate the evaporation rate of water.

Moreover, under the control of ET_p_, the monthly average temperature, precipitation, average pressure, average relative humidity, air density, average wind speed, and solar radiation were analyzed for correlations and partial correlations in the basin over the past 30 years. The upper right portion of Table 3 shows the partial correlation analysis coefficients and the lower left portion shows the correlation analysis coefficients (Table 3). Except for precipitation and average wind speed, the other meteorological factors and ET_p_ passed the confidence test of 0.05.

**Table 3:** Principal component analysis load matrix of meteorological elements in Hancang river basin.

Ingredient	Principal component 1	Principal component 2	Principal component 3
Average temperature (℃)	0.97650	0.08352	− 0.17556
Precipitation (mm)	0.92084	− 0.00271	0.38409
Average pressure (kPa)	− 0.96041	− 0.23987	0.03647
Average relative humidity (%)	0.68003	− 0.71225	0.08266
Air density (kg/m^3^)	− 0.98106	− 0.09487	0.14966
Average wind speed (m/s)	− 0.29823	0.19466	0.71491
Solar radiation (W/m^2^)	0.90340	0.39915	− 0.02473

Table 4 shows that the correlation coefficient between solar radiation and ET_p_ is the highest and is followed by average temperature and precipitation. Average air pressure and air density are significantly negatively correlated with ET_p_. Partial correlation coefficients show the interactions of various meteorological factors after eliminating the influence of ET_p_ factors. The correlation between average wind speed and precipitation is not significant; average air pressure and air density are negatively correlated with precipitation; and average temperature, relative humidity, solar radiation and precipitation are positively correlated. Average wind speed is negatively correlated with all meteorological factors except for precipitation. The correlation between average relative humidity and precipitation is the strongest. The analysis shows that the average relative humidity is greatly affected by ET_p_.

**Table 4:** Correlation analysis between meteorological factors and ET_p_.

Variable	Average temperature	Precipitation	Average pressure	Average relative humidity	Air density	Average wind speed	Solar radiation
Average temperature		0.093**	− 0.005**	0.453**	− 0.989**	− 0.504**	0.223**
Precipitation	0.834**		− 0.345**	0.464**	− 0.175**	− 0.266	0.128**
Average pressure	− 0.955**	− 0.864**		− 0.158**	0.104**	− 0.234**	− 0.514**
Average relative humidity	0.594*	0.651*	− 0.482*		− 0.495**	− 0.915**	− 0.686**
Air density	− 0.999**	− 0.846**	0.965**	− 0.593*		− 0.502**	− 0.230**
Average wind speed	− 0.226**	− 0.239	0.067**	− 0.836**	0.212*		− 0.620*
Solar radiation	0.911**	0.824**	− 0.963**	0.313**	− 0.918**	0.081**	
ET_p_	0.951**	0.844**	− 0.980**	0.520**	− 0.980**	0.116**	0.974**

*Significantly correlated at the 0.05 level.

**Significantly correlated at the 0.01 level.

### Analysis of the changes of surface dry and wet conditions

**Figure 4:**
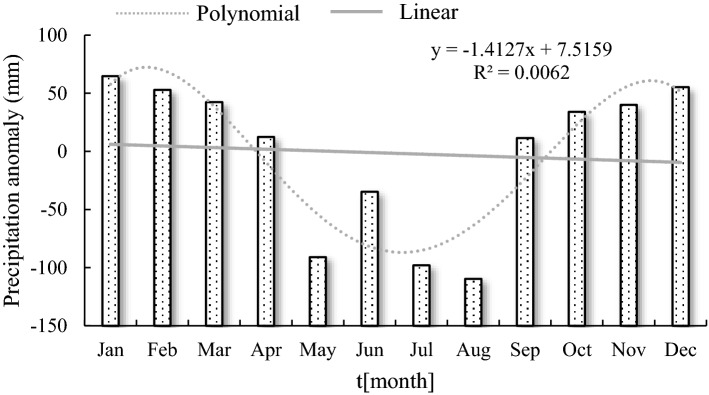
Monthly change trend of precipitation anomaly in Hancang River Basin.

**Figure 5:**
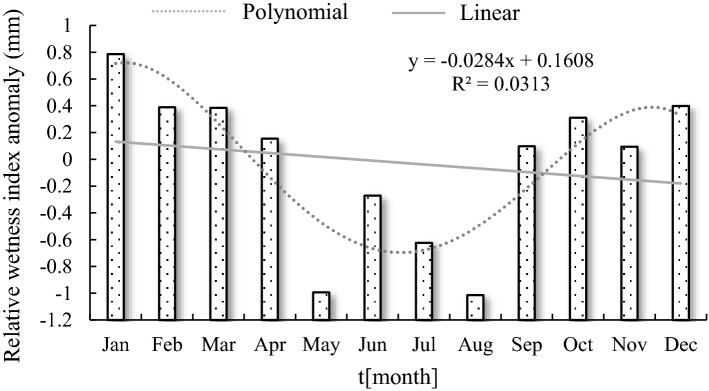
Monthly relative humidity index anomaly in Hancang River Basin.

A large gap in seasonal precipitation in the Hancang River Basin and monthly precipitation show a trend of first increasing and then decreasing with a linear trend rate of − 1.48. Precipitation is mainly concentrated in the flood season in the basin. The maximum monthly precipitation is 174.93 mm (August); the minimum is 0.47 mm (January); and the average monthly precipitation is 65.27 mm. The occurrence of negative anomalies is concentrated in May–August (Fig. 4). The relative wetness index characterizes surface dryness and wetness. The relative humidity index anomaly trend is consistent with precipitation. The maximum value of the monthly relative humidity index is 0.822 (August); the minimum value is − 0.979 (January); and the monthly average value is − 0.193 (Fig. 5).

## Discussion

FAO-56 Penman–Monteith (PM) is a standard model for calculating basin evapotranspiration^34^. This model is based on a rigorous reasoning process and is the classic method for calculating evapotranspiration within a river basin. It is a semi-empirical model. Different regions have different meteorological and hydrological elements and need to be tested and evaluated at specific locations to improve the accuracy of model predictions^35–37^. In addition, there are more suitable evapotranspiration models to estimate evapotranspiration in the study area. Zahra conducted a statistical analysis of different evapotranspiration models and screened evapotranspiration models for their suitability in different arid regions^38^. Feng et al. proposed the Hargreaves (HG) model with temperature as the main reference and conducted an empirical analysis which recommended the HG model as a PM alternative method for evapotranspiration estimations when the dataset required by the PM model is not fully available^39^. Tasumi provided a mapping evapotranspiration with internalized calibration (METRIC) ET estimation model that was adopted to estimate monthly evapotranspiration for a period from the western part of the Urmia Lake Basin, Iran, This method combines basin evapotranspiration data with spatio-temporal resolution and accuracy information and provides more accurate and detailed of evapotranspiration data^40^. Therefore, suitable alternative methods should be selected in different regions, which can accurately assess the relationship between evapotranspiration and meteorological elements and the change law of surface dry and wet conditions.

At present, there are few studies on ET_p_ and relative humidity at small-basin scales. This paper analyzes meteorological observation data from the Hancang River Basin and concludes that precipitation has increased sharply since May (in each year) over the study period and has suddenly decreased the value of the precipitation anomaly value and relative humidity index. The reason may be the enhanced summer circulation in East Asia which has led to increased precipitation^41^; in September, the warm and humid airflow brought by the southeast monsoon has weakened and the amount of precipitation has decreased. In addition, global warming has led to an increase in extreme weather which also directly affects precipitation in the basin. Therefore, it is speculated that precipitation is the main factor which affects summer evapotranspiration in the basin, which is the same as the results of Luab^22^. It is worth noting that in the context of global warming, the average temperature has not the highest impact on ET_p_. The reason may be that the decrease in solar radiation, wind speed and relative humidity overcomes the enhancement of temperature increase on evapotranspiration in the basin. This is the phenomenon of evaporation paradox^42,43^.

The relationship between meteorological factors and ET_p_ is complex and there have been many research advances. Guan et al. adopted the Mann–Kendall test method to detect the change characteristics of ET_p_ in the watershed and concluded that net radiation (Rn), relative humidity (RHU), wind speed (WIN) and temperature (T) are the factors that affect ET_p_^44^. Zhang et al. pointed out that the decrease in wind speed and relative humidity and increase in average temperature have led to a decrease in evapotranspiration levels of the basin^45^. Gao et al. demonstrated that the decreasing solar radiation plays the most important role in the ET decrease in the whole basin, Air temperature followed by relative humidity, and wind speed are the other three main dominating variables ^25^. The above conclusions are consistent with the conclusions of this study. This study points out that average wind speed is one of the important factors affecting ET_p_; Wind speed is one of the most important meteorological factors in aerodynamics, it affects the evaporation rate of water which in turn affects evapotranspiration in the basin. Odongo et al. believe that the direct factor affecting evapotranspiration is solar radiation. Due to the decrease in net solar radiation and increase in actual vapor pressure, temperature differences between the surface and air have decreased, which in turn have reduced evapotranspiration in the basin^46^. Their study concluded that the strongest positive correlation was between solar radiation and ET_p_, and our research supports this view.

The ET_p_ process is closely related to the hydrological process and is affected by many ecological factors within the basin^47^. This article focuses on the preliminary analysis of the time characteristics of evapotranspiration and the reasons for the changes in evapotranspiration, but only a single meteorological factor is considered and cannot directly explain the drought situation in the basin. The climate change impact on evapotranspiration is very complex. If meteorological factors are combined with topographical factors, vegetation changes, soil moisture and human activities, we will arrive at a more accurate and comprehensive understanding of the changing laws of surface dry and wet conditions in the study area^48^.

## Conclusions

Based on the presented results, the following conclusions can be made:

The annual ET_p_ in the basin is 784.143 mm, and the monthly average is 65.345 mm. The overall trend is increasing first and then decreasing, and there are multi-scale time–frequency changes. ET_p_ varies greatly among different months. Wavelet analysis demonstrates that there are 3 clusters or dispersive centers within each year that take place in January, July and December, and the scale of influence is concentrated from 6 to 12.

ET_p_ changes in river basins are the result of the comprehensive effects of various meteorological factors. Average temperature, precipitation, average relative humidity, and solar radiation are positive factors that affect ET_p_ while average air pressure is a negative factor that affects ET_p_. ET_p_ for each meteorological factor has a large response to the average temperature. ET_p_ reacts to meteorological factors to a certain extent.

In exclude ET_p_ control, correlations between air density, solar radiation and average wind speed decrease; correlations between average temperature, precipitation, and relative humidity increase in a positive direction; and for average air pressure, average temperature, air density, and average wind speed, the correlations increase in the negative direction. The average relative humidity is greatly affected by ET_p_.

Monthly precipitation varies greatly in the Hancang River Basin. Affected by seasonal precipitation, both ET_p_ and the relative humidity index show obvious fluctuation trends and both have significant positive correlations with precipitation. The surface wetness of the basin presents a state of drought–humidity–drought with the relative humidity index.

## Data Availability

Transparency.
